# The WRITTEN-HEART study (expressive writing for heart healing): rationale and design of a randomized controlled clinical trial of expressive writing in coronary patients referred to residential cardiac rehabilitation

**DOI:** 10.1186/1477-7525-9-51

**Published:** 2011-07-08

**Authors:** Gian Mauro Manzoni, Gianluca Castelnuovo, Enrico Molinari

**Affiliations:** 1 Istituto Auxologico Italiano IRCCS, Psychology Research Laboratory, Ospedale San Giuseppe, Verbania, Italy; 2 Department of Psychology, University of Bergamo, Bergamo, Italy; 3 Department of Psychology, Catholic University of Milan, Milan, Italy

## Abstract

**Background:**

Coronary heart disease (CHD) is typically associated with many cardiovascular risk factors (e.g., elevated blood pressure), low health-related quality of life, depression, anxiety and psychological stress. Expressive writing (EW) has shown beneficial effects on such variables in both people from the community and in patients with a variety of chronic illnesses. However, no study to date has evaluated the physical and psychological effects of the expressive writing procedure on coronary patients referred to cardiac rehabilitation (CR).

**Methods:**

The clinical effectiveness of a 2-week disease-related expressive writing procedure (writing about one's deepest thoughts and feelings regarding the experience with heart disease) compared with the standard writing task (writing about one's deepest thoughts and feelings about the most traumatic or negative event experienced in the life), a neutral writing condition (writing about the facts regarding heart disease and its treatment) and an empty control condition will be evaluated in a randomized controlled clinical trial (RCT) with repeated follow-up measurements at 3, 6 and 12 months after discharge from CR. The primary outcome is health-related quality of life (SF-12). Secondary outcome measures are depression (BDI-II), anxiety (BAI) and post-traumatic growth (PTGI). Furthermore, the study will explore the moderating effects of coping styles, type D personality, perceived emotional support and participants' evaluative ratings of the writing interventions on the main experimental effects in order to identify sub-groups of patients showing different results.

**Discussion:**

The WRITTEN-HEART study aims to explore and expand the frontiers of the expressive writing research enterprise by investigating the feasibility, safety and clinical efficacy of brief and cost-effective expressive writing interventions in patients with CHD referred to CR.

**Trial registration:**

ClinicalTrials.gov NCT01253486

## Background

The World Health Organization (WHO)'s statistics suggest that cardiovascular diseases (CVDs) are the number one cause of mortality for all males and females, accounting for almost 30% of all global deaths [1].

Despite this alarming scenario, the age-adjusted cardiovascular (CV) death rates have decreased almost 50% during the past 25 years [2]. Improved management of acute myocardial infarction (MI), earlier diagnostic procedures, advanced intervention techniques and especially the recognition and management of CV risk factors have resulted in an increasing number of CVD survivors [3]. However, such gains in survival rates have resulted in a significant increase in physical disability, impaired health-related quality of life and high psychosocial distress, particularly in the rapidly growing population of elderly persons [4,5].

Psychosocial and affective disorders are highly prevalent within cardiac populations and contribute significantly to impair health-related quality of life and also to enhance the prognostic risk for adverse CV events [5]. For example, the prevalence rate of major depression disorder in the National Comorbidity Survey [6] was approximately 5%, compared with a prevalence of 15% or greater in populations with CVD [7]. In addition, at least another 15% to 20% of patients with cardiac disease exhibit depressive symptoms that do not necessarily meet criteria for major depressive disorder [7]. Similarly, anxiety disorders, such as phobic anxiety or panic disorder, are relatively common among patients with CVD. As a consequence, cardiologists are likely to encounter a significant number of psychologically distressed patients in medical practice [5]. Clinical health psychologists have thus an important role in helping to develop effective psychosocial interventions for this population [8,9].

### Study rationale

A number of controlled experiments have demonstrated the physical and psychosocial benefits of expressive writing. Developed by Pennebaker and Beall [10], the procedure typically involves random assignment of individuals to one of two conditions, with instructions to write about their deepest thoughts and feelings regarding a stressful experience or about innocuous topics (control) over several brief writing sessions.

A recent special issue of the British Journal of Health Psychology edited by Joshua Smyth and James Pennebaker [11] confirms the breath of current interest in the expressive writing paradigm and invites to look forward at the many remaining frontiers in Expressive Writing research. One of the boundary conditions that have been identified in their commentary paper concerns new outcome measures and previously unexamined populations. Surprisingly, the expressive writing procedure has never been used with patients with CVD. Perhaps, clinical researchers have been negatively impressed by some negative results [12]. Furthermore, in a review on the putative theories underlying the expressive writing procedure, Sloan and Marx concluded that it was too early to say definitively whether expressive writing is a trustworthy technique that should be adopted by the therapeutic community [13]. However, the meta-analysis by Frisina, Borod and Lepore [14] on 9 writings studies using clinical populations showed that expressive writing significantly improved health outcomes (d = .19) and the strongest effect was found for physical health outcomes (d = .21). Moreover, the expressive writing procedure is tremendously cost-effective, easy to administrate, feasible and brief, does not need highly trained clinicians to work, has a great accessibility and has shown an amount of promising objective and subjective health benefits in many studies on college students and individuals from the community. Such appealing features have made it very attractive for some clinicians and clinical researchers who, beginning in the 90s and going on until nowadays, have started to use the expressive writing procedure with their patients and to examine its effects empirically in many randomized clinical trials involving individuals with physical disorders such as breast cancer [15-18], rheumatoid arthritis [19], fibromyalgia [20], HIV [21,22], renal cell carcinoma [23], men diagnosed with prostate cancer [24], women with chronic pelvic pain [25], men with elevated blood pressure [26], patients undergoing bladder papilloma resection [27] and transurethral prostate resection [28].

The fact that no trial to date has yet evaluated the effects of expressive writing on patients with CVD is surprising because many studies on the physiological effects of expressive writing have found significant and beneficial variations in many markers of the autonomic nervous system such as skin conductance, heart rate, heart rate variability and blood pressure [e.g., [17]]. It is even more disappointing that the small but significant study of McGuire, Greenberg and Gevirtz [26] on the autonomic effects of expressive writing in individuals with elevated blood pressure has been neither replicated nor expanded in the subsequent years. In their paper, McGuire, Greenberg and Gevirtz argued that, given the high costs and potential risks of elevated blood pressure and the lack of demonstrated effective non-pharmacological treatments for this population, a low-cost and easily administered psychological intervention as expressive writing, if shown to be effective, has the potential for widespread clinical use. Although they showed that, one month after writing, the participants who were allocated in the expressive writing condition exhibited lower systolic and diastolic blood pressure than before writing and that, four months after writing, diastolic blood pressure remained lower than baseline levels, their argument felt on deaf ears. As elevated blood pressure is a major neuro-cardiovascular risk factor that often affects patients with an established CVD, an intriguing idea is to administer the expressive writing procedure to a sample of patients with CVD referred to cardiac rehabilitation (CR) and test the brief, mid and long-term effects of such an intervention on patients' health-related quality of life (HRQoL), anxiety symptoms, depressive symptoms, medical consultations for cardiovascular morbidity and post-traumatic growth. With respect to the latter outcomes, same evidence suggests that expressive writing is effective in enhancing positive growth from trauma over time [29] and a body of research has shown that awareness of the benefits of adverse events and circumstances is an important predictor of successful adjustment [30-32].

Further empirical support to the rationale of this study comes from the results of a recent clinical trial on potential physiological, emotional and cognitive mechanisms underlying the positive health effects produced by disease-related expressive writing in a sample of women with early stage breast cancers [17]. Findings supported the hypothesis that autonomic activity (heart rate) mediates the effect of the expressive writing condition on self-reported physical symptoms. Findings suggested that the prolonged and repeated exposure and concomitant cognitive processing might contribute to improved regulation of physiological responses, presumably leading to less stress on bodily systems and ultimately enhanced physical health [17].

A second goal of the study is to determine whether the effects of the expressive writing intervention vary as a function of four potential moderating variables: coping styles, negative affectivity and social constraint (type D personality factors), perceived social support and evaluative ratings of the writing intervention. In fact, some evidence suggests that expressive writing may be most effective for individuals who use more approach-oriented, expressive coping strategies than for those who are more non-expressive or have deficits in identifying and processing emotion [33-35]. Further, same evidence suggests that perceived emotional support is a moderator of the expressive writing effects. This hypothesis was guided by social constraint theory, which suggests that the absence of social outlets for emotional expression and processing has a negative effect on adjustment to stressful situations and that expressive writing may represent a useful intervention for individuals who lack opportunities for emotional expression in their social environments [36].

## Methods

### Study design

A four-arm randomized controlled clinical trial with four follow-up assessments (immediately before discharge from hospital, 3 months, 6 months and 12 months after discharge) will be carried out in order to test the following primary hypotheses/outcomes: 1) a modified disease-related expressive writing intervention is effective in enhancing physical and psychological health outcomes (HRQoL, anxiety and depression symptoms, medical visits for CVD-related morbidities) relative to a sham condition in which patients write solely about the facts of their experience with CVD and relative to a control empty condition; 2) the modified disease-related expressive writing intervention is more effective in enhancing physical and psychological health outcomes than a standard expressive writing condition in which patients write about the their deepest thoughts and feelings about the most traumatic or negative event they have experienced in their life. Further hypotheses/outcomes concern post-traumatic growth and are: 1) the modified disease-related expressive writing intervention is effective in enhancing post-traumatic growth relative to sham and control conditions; 2) the modified disease-related expressive writing intervention is more effective in enhancing post-traumatic growth than the standard expressive writing task.

Secondary analyses will be conducted in order to explore the relative efficacy of the writing conditions as a function of patients' gender, age, coping styles, negative affectivity and social constraint (type D personality factors), perceived social support and evaluative ratings of the writing intervention. Accordingly, significant interactions between the experimental conditions and the moderator variables mentioned above are postulated such that, for example, patients low on avoidance will benefit more from expressive writing than avoidant patients and that patients perceiving low social support will benefit more than patients perceiving high social support.

The Medical Ethics Committee of Istituto Auxologico Italiano approved the study protocol.

### Study population

#### Recruitment of participants and selection criteria

All coronary patients who will be referred to the S. Giuseppe Hospital of the Istituto Auxologico Italiano for residential cardiac rehabilitation (CR) and who will meet inclusion criteria of having had a medical diagnosis of Coronary Heart Disease (CHD) and being affected by major cardiovascular risk factors will be asked and screened for admission in the study. CHD is defined as a history of at least one of the following conditions: myocardial infarction, coronary artery by-pass grafting (CABG) and coronary angioplasty (PTCA). Patients will not be selected if they will be diagnosed with recent (less than four weeks) myocardial infarction, CABG or PTCA, if they will be unable to read and write in Italian and if they will have an age ≥ 70 years.

### Randomization procedure and blinding

All participants will be randomly assigned to the experimental conditions in a consecutive way. The simple randomization scheme will be generated by using the Web site http://www.randomization.com. Random allocation will take place after the baseline measurements and patients will be blind to condition assignment.

### Sample size calculation

Given that no study has yet evaluated the health effects of expressive writing on patients with CVD and considering the small mean effect sizes calculated by Frattaroli in her meta-analysis on expressive writing studies [37], no appropriate and reasonable empirical data are available for calculating the necessary sample size that would allow a high chance to detect a significant difference across conditions. Because of this, the study may be considered partially explorative and a very large sample (the one necessary to detect the small effect sizes obtained by Frattaroli in her meta-analysis) is thus not needed. Hence, calculations were based on the explorative assumption that the effect of expressive writing on coronary patients' health-related quality of life is large (f = 0.4) in accordance to Cohen's classification [38]. We further decided not to make assumptions on sidedness because the expressive writing procedure may also have negative effects in comparison with the control empty group. On the basis of such considerations, a total sample of 92 participants (n = 23 for each condition) is required taking into account a total dropout rate of 20% to detect this large difference with an alpha of 0.05 two-sided and a power of 0.82. Calculations were made with SamplePower (Release 2.0; SPSS, Inc., Chicago, IL).

### Experimental and clinical protocol

Patients will be recruited from a residential cardiovascular rehabilitation unit in the northwestern of Italy. The cardiac rehabilitation (CR) program lasts 1 month for each patients in accordance with the Italian Health Institute's guidelines. Along this period, patients live in the S. Giuseppe hospital, which is located on a mountain highland and far away from towns and cities. Few days after entry in the CR unit and immediately after the initial medical evaluation and treatment planning (first week), patients will be approached by the research investigator for initial screening in accordance to inclusion and exclusion criteria. Patients who will go through the screening will be informed orally by the research investigator that a scientific study is ongoing and that its purpose is "to learn more about how individuals adjust to having heart disease". They will be also told that they could be asked to write about their experiences with heart disease and, if they will consent, they will be scheduled for the following day when they will receive the informed consent form, which includes no mention of expected benefits from the writing sessions and no mention of the randomization to one of four conditions. Once patients will have signed the informed consent form, they will be administered the baseline questionnaires. With the exception of the control participants, they will receive a schedule relative to the four writing sessions to be completed within the following two weeks. Once randomization will be performed, no change in treatment allocation will take place in the future. Participants will complete the individual writing sessions in a peaceful laboratory close to the CR unit. Writing instructions will be written on the sheets that patients will use to write and will be presented to them at the beginning of each writing session. A research assistant will meet with each participant just before writing, will give him the writing sheets and will let him alone in the laboratory for twenty minutes, then she will return to stop the session and to pick up the sheets.

Participants will be randomized to one of the following four conditions: 1) disease-related expressive writing (DS-EW); 2) standard expressive writing (S-EW); 3) unemotional writing (Sham) and 4) an empty condition not involving a writing task (assessments only). All the active conditions (1, 2 and 3) will consist in four 20-minute writing sessions that will occur during the hospital stay within a 2-week period and that will be scheduled twice a week for each participant.

Writing instructions for the three active groups will be as follows:

At the end of the CR program and before discharge, all participants, including those assigned to the empty control condition, will be re-administered the outcome questionnaires and will be told that they will receive other follow-up questionnaires by mail at 3, 6, 9 and 12 months after discharge from hospital. A form on which to record any medical visits or events over the previous 3 months will be also sent at each participant at each follow-up assessment.

#### 1. Disease-related expressive writing (DS-EW)

"What I would like you to write about for these four sessions are your deepest thoughts and feelings about your experience with heart disease. I realize that individuals with heart disease experience a full range of emotions and I want you to focus on any and all of them. In your writing, I want you to really let go and explore your very deepest emotions and thoughts. You might think about all the various feelings and changes that you experienced before being diagnosed, after diagnosis, during treatment, and now. Whatever you choose to write, it is critical that you really focus on your deepest thoughts and feelings. Ideally, I would like you to focus on feelings, thoughts or changes that you have not discussed in great detail with others. You might also tie your thoughts and feelings about your experiences with heart disease to other parts of your life - your childhood, people you love, who you are or who you want to be. Again, the most important part of your writing is that you really focus on your deepest emotions and thoughts. The only rule we have is that you write continuously for the entire time. If you run out of things to say, just repeat what you have already written. Don't worry about grammar, spelling or sentence structure. Don't worry about erasing or crossing things out. Just write."

#### 2. Standard expressive writing (S-EW)

"What I would like you to write about for these four sessions are your deepest thoughts and feelings about the most traumatic or negative experiences you had in your life. I realize that individuals who live a traumatic experience have a full range of emotions and I want you to focus on any and all of them. In your writing, I want you to really let go and explore your very deepest emotions and thoughts. Whatever you choose to write, it is critical that you really focus on your deepest thoughts and feelings. Ideally, I would like you to focus on feelings, thoughts or changes that you have not discussed in great detail with others. You might also tie your thoughts and feelings about your negative experiences to other parts of your life - your childhood, people you love, who you are or who you want to be. Again, the most important part of your writing is that you really focus on your deepest emotions and thoughts. The only rule we have is that you write continuously for the entire time. If you run out of things to say, just repeat what you have already written. Don't worry about grammar, spelling or sentence structure. Don't worry about erasing or crossing things out. Just write."

#### 3. Unemotional writing (Sham)

"What I would like you to write about for these four sessions is a detailed account of facts regarding your heart disease and its treatment. I am interested in how the specifics of detection, diagnosis and treatment differ among individuals with heart disease; therefore, it is critical that you provide an extremely detailed account of all that happened to you with regard to having heart disease. I realize that individuals with heart disease experience many emotions, but in your writing I want you to focus only on the facts, not on your emotions. No fact is too big or too small. You might write about when your hear disease was discovered and who discovered it, appointments that you had with doctors or other people about your heart disease, information you were given and what treatment was chosen. You might recount your experience from beginning to present day, including all the factual details you can think of. Again the most important part of your writing is that you focus on the facts and try to reconstruct what happened in as great factual detail as possible. The only other rule ... [Instructions continue as above]

### Outcome Measures

#### SF-12

The SF-12 Health Survey is a measure of physical and mental health. It is the short form of the most popular SF-36 and consists of the 12 items that were found to be the best predictors of the two SF-36 physical and mental summary scores (referred to as PCS-36 and MCS-36, respectively) in the US validation study [39]. Selected items and weights derived from the general US population were then used to score the physical and mental summary scores (referred to as PCS-12 and MCS-12, respectively). The PCS-12 and MCS-12 were very highly correlated with PCS-36 and MCS-36 (r = 0.951 and 0.969, respectively) and were very weakly correlated (r = 0.06) with each other in the US sample. In the present study, the Italian version of the SF-12 will be used [40] and the two summary scores (PSC-12 and MCS-12) will be computed with weights derived from the Italian validation sample.

#### Beck Depression Inventory - II

The Beck Depression Inventory - Second Edition (BDI-II) is a measure of depressive symptoms. It was developed by revising the BDI in response to the American Psychiatric Association's publication of the Diagnostic and Statistical Manual of Mental Disorders, Fourth Edition, which changed many of the diagnostic criteria for Major Depressive Disorder. Indeed, the BDI items involving changes in body image, hypochondria and difficulty working were replaced. Also, sleep loss and appetite loss items were revised to assess both increases and decreases in sleep and appetite. All but three of the items were reworded; only the items dealing with feelings of being punished, thoughts about suicide and interest in sex remained the same. Finally, respondents rate how they have been feeling for the past two weeks, as opposed to the past week as in the original BDI. Like the BDI, the BDI-II also contains 21 items and each answer is scored on a scale that ranges from 0 to 3. A total score is computed by summing all the ratings and higher total scores indicate more and more acute depressive symptoms. The Italian study to establish the validity and reliability of the measure indicated that the BDI-II is positively correlated with the Hamilton Depression Rating Scale. The test was also shown to have a high one-week test-retest reliability (Pearson r = 0.93), suggesting that it was not overly sensitive to daily variations in mood. The test also has high internal consistency (α = .91).

#### Beck Anxiety Inventory

The Beck Anxiety Inventory (BAI) is a measure of anxiety symptoms. It consists of 21 items that represent 21 psychological or somatic symptoms of anxiety (such as numbness, hot and cold sweats or feelings of dread). The respondent is asked to rate on a scale which ranges from 0 (*Not at all*) to 4 (*SEVERELY: I could barely stand it*) how each symptom has caused him distress in the previous week. A total score is computed by summing all the ratings and higher total scores indicate more and more severe anxiety symptoms.

#### Post-Traumatic Growth Inventory - Short Form

The Post-Traumatic Growth Inventory - Short Form (PTGY-SF) was derived analytically from the 21-item PTGY [41] and consists of the 10 items that loaded most on each of the five underlying factors [42]. In particular, the items with the highest loadings on each factor were examined and the two with the highest loadings were selected for three (Spiritual Change, Appreciation of Life and Personal Strength) of the five factors. For the remaining two factors (Relating to Others and New Possibilities) the two items with the highest loadings were not selected because they were too redundant in content; instead items were selected in order to improve the breadth of coverage. In the US validation study, the 10-item PTGI-SF had internal reliability only very slightly lower than the full form PTGI, and the reliability of the total score was generally in the range of .90 across a variety of samples [42]. In the present study, the Italian translation of the PTGY-SF 10 items were used [43] and a total score for each participant was computed by averaging the responses to all the items.

#### Frequency of medical visits for cardiovascular morbidity

A form on which to record any medical visits over the previous 3 months will be also sent at each participant at the four follow-up time points. Patients have to retrospectively record all medical visits during the previous 3 months. They have to record also the medical provider and the reason for each visit (e.g., check-up with medical cardiologist). These medical appointments will be coded as a function of reason for the visit (i.e., routine and non-routine CVD-related and non-CVD-related appointments) by the research investigator who will be aware of participants' condition assignment. Medical appointments for CVD-related problems will be of interest, excluding scheduled check-ups, as an indicator of morbidity associated with CVD and its treatment. The other categories of medical appointments (i.e., CVD-related scheduled medical check-ups, other scheduled medical check-ups or non-routine medical appointments for other problems, such as flu symptoms) will be combined for analysis.

### Covariates

#### CVD-related past and actual perceived stress

Along with the collection of demographic and clinical data and before the questionnaires' administration, patients will be asked to rate how much stressful was the CVD onset and how much stressful is currently the CVD on seven-point scales from (1) not at all to (7) extremely.

#### Writing ratings

Immediately following the last writing session, participants will be asked to rate sixteen items reflecting some aspects of the writings and of the whole writing experience on seven-point scales from (1) not at all to (7) extremely. For example, participants will be asked to rate how emotional and how personally meaningful the essays were.

#### Self-reported mood

Immediately prior to and after each writing session, participants will complete a restricted and "right now" version of the Profile of Mood States (POMS). The scale has 65 affect adjectives rated on a 7-point scale (0 not at all, 7 extremely). As in other studies [17,34,44], we will construct a distress index (Distress) by adding items (e.g., tense, sad) on the Anger, Depression, Tension, Fatigue and Confusion subscales, and we will use the Vigor subscale (e.g., energetic, cheerful) to indicate positive mood.

#### Type D Personality Inventory

The Type D Personality Scale (DS14) is a 14-item scale comprising of two subscales [45]: a seven-item subscale which measures negative affectivity (NA) (e.g., "I often feel unhappy") and a 7-item subscale measuring social inhibition (SI) (e.g., "I often feel inhibited in social interactions"). Respondents rate their personality on a five-point Likert-type scale which ranges from 0 = false to 4 = true (Items 1 and 3 were reverse scored). The NA and SI scales can be scored as continuous variables (range, 0-28) to assess these personality traits independently. Participants who score highly on both NA and SI using a cutoff point of ≥10 on both scales are classified as having a Type D personality.

#### Brief COPE

The Brief COPE is a self-completed questionnaire measuring coping strategies [46]. It is the short form of the most famous 60-item COPE inventory (15 scales with 4 items per scale) and consists of 28 items that compose 14 scales of two items each. Two scales from the full measure were omitted from the brief form because they did not proven useful in previous work or had proven redundant with another scale (Restraint Coping and Suppression of Competing Activities). Three other scales were refocused slightly because they had proven to be problematic in previous work. Positive Reinterpretation and Growth became Positive Reframing (omitting any mention of growth), Focus on and Venting of Emotions became Venting (omitting items that had appeared to relate too closely to experiencing distress) and Mental Disengagement became Self-Distraction. A new scale (self-blame) - not part of the original COPE - was added because of evidence of its importance [46]. Respondents rate each item on a four-point Likert scale which ranges from 1 (*I usually do not this at all*) to 4 (*I usually do right like this*). Items and response options can be converted to a dispositional "coping style" format (the one I used for the present study) and to a situational concurrent format. The first study to establish the validity and reliability of the measure indicated that the a priori scales had adequate internal reliability (from α = 0.82 for Religion to α = 0.54 for Denial) and that the factor structure was generally consistent with that reported for the full COPE [46]. In the present study, the Italian version of the Brief COPE will be used [47] and the 14 scale scores will be computed by averaging the responses to the two composing items.

### Experimental manipulation check

An independent rater not involved in the study and unaware of condition membership will judge whether each writings, randomly ordered, will conform to condition instructions.

### Statistical analysis

Data entry will be conducted by a trained research assistant who will be aware of participants' condition assignment but not of the research hypotheses. Preliminary analyses will be performed to examine assumptions for parametric statistical analysis and baseline equivalence among groups. Given the longitudinal nature of the data (repeated measurements), multilevel modeling analysis in MLwiN 2.21 (Centre for Multilevel Modeling-CMM, University of Bristol) will be used to study health outcomes (physical and mental health, anxiety, depression, post-traumatic growth and number of medical visits for cardiovascular morbidity) as functions of time and experimental conditions. This will allow testing of experimental hypothesis as cross-level interactions between slopes representing time (1^th ^level) coded as 0 for the first (immediately before discharge from hospital) follow-up, as 1 for the second (3-month) follow-up, as 2 for the third (6-month) follow-up and as 3 for the fourth (12-month) follow-up, and a categorical covariate representing conditions (2^nd ^level). We will start with a simple random intercept model (for calculating intra-class correlation [ICC]) and a simple linear growth model, followed by a model with experimental conditions as a time-invariant covariate. We then will structure more complicated models, including models with different within-individual covariance structures and models with nonlinear growth patterns. A critical alpha of 0.05 will be considered for hypothesis testing. Furthermore, on the basis of the best multilevel models structured in the primary analyses, we will explore the moderating effects of some putative covariates at level 2 such as gender, age, coping styles, negative affectivity and social inhibition (Type D personality dimensions), perceived social support and writings ratings. For these secondary explorative analyses no level of significance will be defined. Readers interested in further discussion of analyzing longitudinal data with multilevel modeling analysis are referred to an excellent guide by Kwok et al. [48] as well as a thorough treatment of the issue by Singer and Willett [49].

## Discussion

For the first time the expressive writing procedure will be administered to a sample of patients with CVD referred to a 3-week residential cardiac rehabilitation program. The feasibility, safety and clinical efficacy of such a brief psychological intervention will be evaluated in a four-arm randomized controlled clinical trial with a 12-month follow-up period. Multiple psychological variables relevant to patients with CVD will be assessed in order to examine the specific effects of the expressive writing intervention on some psychosocial risk factors related to CV morbidity in this population. Furthermore, a generic measure of health-related quality of life (HRQoL) will be administered to participants at each follow-up assessment as HRQoL improvement is among the major outcomes of clinical trials [50] and was established as one of the primary goals of cardiac rehabilitation [51].

Given the novelty of the study and our will to handle a low number of outcomes, physiological data such as heart rate, blood pressure and cholesterol will not be considered for the analyses and only a measure of medical visits or events along the follow-up period will be used as a surrogate marker of bio-medical health.

This study is essentially intended to explore and expand the clinical frontiers of the expressive writing research enterprise. Altough no advancement in the theorical knowledge about the multi-level mechanisms that underlie the expressive writing task is scheduled, the results of this trial will contribute to the evidence-based knowledge on the application of the expressive writing procedure for clinical purposes in clinical settings [52].

## Competing interests

The authors declare that they have no competing interests.

## Authors' contributions

GMM conceived the study, planned its design and made substantial contribution to the manuscript drafting. GC participated in the study design and contributed to the manuscript drafting. EM participated in the study design and helped to draft the manuscript. All authors read and approved the final manuscript.
